# Stress induced proinflammatory adaptations: Plausible mechanisms for the link between stress and cardiovascular disease

**DOI:** 10.3389/fphys.2023.1124121

**Published:** 2023-03-17

**Authors:** Aaron L. Slusher, Edmund O. Acevedo

**Affiliations:** ^1^ Department of Pediatrics, Yale University School of Medicine, New Haven, CT, United States; ^2^ Department of Athletics, Yale University, New Haven, CT, United States; ^3^ Department of Kinesiology and Health Sciences, Virginia Commonwealth University, Richmond, VA, United States

**Keywords:** stress, allostasis, inflammation, cardiovascular disease, physical activity

## Abstract

Initiating from Hans Selye’s conceptualization of stress physiology, to our present understanding of allostatic load as the cumulative burden of chronic psychological stress and life events, investigators have sought to identify the physiological mechanisms that link stress to health and disease. Of particular interest has been the link between psychological stress and cardiovascular disease (CVD), the number one cause of death in the United States. In this regard, attention has been directed toward alterations in the immune system in response to stress that lead to increased levels of systemic inflammation as a potential pathway by which stress contributes to the development of CVD. More specifically, psychological stress is an independent risk factor for CVD, and as such, mechanisms that explain the connection of stress hormones to systemic inflammation have been examined to gain a greater understanding of the etiology of CVD. Research on proinflammatory cellular mechanisms that are activated in response to psychological stress demonstrates that the ensuing low-grade inflammation mediates pathways that contribute to the development of CVD. Interestingly, physical activity, along with its direct benefits to cardiovascular health, has been shown to buffer against the harmful consequences of psychological stress by “toughening” the SAM system, HPA axis, and immune system as “cross-stressor adaptations” that maintain allostasis and prevent allostatic load. Thus, physical activity training reduces psychological stress induced proinflammation and attenuates the activation of mechanisms associated with the development of cardiovascular disease. Finally, COVID-19 associated psychological stress and its associated health risks has provided another model for examining the stress-health relationship.

## Introduction

The term s*tress* is used indiscriminately in modern culture to describe psychological and physiological experiences resulting from the ways in which we live, work, and coexist as members of an increasingly more complex society. *Stress*, and our understanding of the experience, stems in part from the “general-adaptation-syndrome” first proposed by Hans Seyle (1907–1982) in his seminal *Nature* publication over 85 years ago (Seyle, 1936). Over the course of roughly 40 years, Selye built upon the *internal milieu* concept described by Claude Bernard (Bernard, 1879) and Walter Cannon’s homeostatic “alarm phase” in response to emotional and physical duress that involves the release of catecholamines, epinephrine (EPI) and norepinephrine (NOR), from sympathetic nervous system activation of the adrenal medulla [sympathetic adrenal medullary (SAM) system] during a “fight or flight” response (Cannon, 1914a; 1932). In doing so, Selye redefined our understanding of how the body maintains homeostasis in response to a stressor. Additionally, Seyle provided the framework necessary to detail the intricate synchronization of the SAM system with the hypothalamic-pituitary-adrenal (HPA) axis and resulting mechanistic actions linked to the release of catecholamines and glucocorticoids, respectively, in response to chronic stress.

Selye and his colleagues conducted extensive research that led to the understanding that the release of corticotropin releasing hormone (CRH; *via* the hypothalamus) induces the secretion of adrenocorticotrophic hormone (ACTH) from the anterior pituitary, which in turn stimulates the release of glucocorticoids from the adrenal cortex. Furthermore, Selye’s findings provided insight to support his proposed theory that chronic stress, such as prolonged exposure to noxious agents (e.g., extreme temperature, trauma, and various drugs), results in “morphological manifestations of distress” characterized by the “stages of resistance and exhaustion” (Selye, 1936; 1976). More specifically, during the *resistance* stage, Selye detailed that the body prepares for a sustained attack while the immune system increases in activation. However, during the *exhaustion* phase*,* the body’s ability to sustain a response to a stressor is diminished and health begins to deteriorate.

As Selye’s work progressed and his knowledge of the physiology of stress developed, his theoretical propositions evolved. For example, his original detailing of the “general adaptation syndrome” included the notion that activation of the HPA axis by a stressor is a “basic reaction pattern irrespective of the agent used to produce stress” (Selye, 1950). In time, this idea progressed to an appreciation that an individual’s stress response to a particular stressor is, in part, determined by “how you react to it.” In doing so, Selye suggested that the differentiation between “distress” and “eustress” helps to determine the response as being initiated by a negative or positive emotion, respectively (Selye, 1974).

Selye’s foundational contribution in the study of the stress response has led to over 1,500 peer-reviewed articles and nearly 40 books related to the subject. Likewise, his relentless advocacy for the acceptance of the term “stress” within the medical community has contributed to the widespread use (and misuse) of “stress” as a general concept. The term is fully integrated within the lexicon of the broader scientific community and into the shared consciousness of the general public. However, his lasting impact may best be observed by the extension and expansion of his original works across a diverse array of research applications within numerous scientific disciplines. Indeed, his collective work served as a foundation for modern fields of psychoneuroendocrinology (PNE) and psychoneuroimmunology (PNI), which collectively, provided a greater appreciation for the psychological aspect of disease pathology.

### Acute and chronic stress and cardiovascular disease

Hans Selye’s original conceptualization of stress physiology and our present understanding of allostatic load as the cumulative burden of chronic stress and life events have helped to elucidate the physiological mechanisms linking stress to health and disease (Sterling and Eyer, 1988). In response to acute psychological stress, the amygdala, a region within the brain involved in emotional regulation, plays a role in the deactivation of the parasympathetic nervous system and activation of the SAM system and HPA axis (Osborne et al., 2020). Although the SAM system is known to secret many factors, including adenosine triphosphate (ATP), neuropeptide Y, and nitric oxide (MacArthur et al., 2011), SAM system activation is most well-known to initiate the rapid release of the catecholamines EPI and NOR that are observed in circulation throughout the body. Within a similar timeframe, CRH released into portal circulation stimulates ACTH secretion to elicit elevations in plasma cortisol (CORT) within ∼15–20 min (Sapolsky et al., 2000). The role of the SAM system in response to stress is vital to prepare the body for a “fight-or-flight” response by increasing heart rate, blood pressure, the diversion of blood flow and metabolic substrate to skeletal muscle and the brain, the regulation of sweat production, and the downregulation of reproduction and digestion (Karemaker, 2017). The half-lives of EPI and NOR are ∼1.5 and 2.5 min (LaBrosse et al., 1961; Hagberg, et al., 1979), respectively, and once the stressor has resolved, EPI and NOR are rapidly cleared from circulation. In contrast, the HPA axis serves as a self-regulator with CORT by inhibiting CRH and ACTH production and acting to maintain and regulate the coordinated stress response until well after the stressor has subsided (Gagner and Drouin, 1985; McEwen, 2000). With a half-life of ∼60 min (Weitzman et al., 1971), the sustained presence of CORT within circulation returns the body’s internal physiological functions to normal (Droste et al., 2008; Ulrich-Lai and Herman, 2009).

The initial “fight-or-flight” response to acute psychological stress was proposed by Walter Cannon to maintain internal stability and functionality of an organism (Cannon, 1914b). Physiological functions, such as pH and body temperature, however, operate within a narrow range of function, and the body’s ability to regulate these functions within these narrow ranges are considered necessary to sustain life. In the 1970s, Selye theorized that adaptive mechanisms create new equilibriums within an organism in response to a potential threat, such as defense against a pathogen or an increase in the tolerance of tissue to the pathogen (Selye, 1973; Selye, 1975). Having termed this mechanism *heterostasis,* Selye suggested that fluidity of the body’s functional capacity is necessary to artificially increase homeostasis to a new level, and by “resetting the thermostat,” equilibrium is reestablished over the long-term through active “exogenous intervention” *via* pharmacological therapies (Selye, 1973; Selye, 1975). As stress research continued to advance, greater emphasis was placed on the understanding that the body can adapt to a sustained or prolonged stressor that is present within an individual’s environment. For example, heart rates, blood pressures, plasma glucose concentrations, and many other vital functions can operate within a broad range and adapt to new set points in a manner dependent on the physical and psychological state of an individual (McEwen and Stellar, 1993). This functional fluidity has been termed “allostasis” and is necessary to readjust the body’s physiology to maintain internal stability in response to change (McEwen, 1998).

During a “normal” acute stress response, allostasis represents a physiological response reflective of the individual’s resilience to the stressor and serves as a temporary protective buffer against a potentially harmful physical or emotional perturbation. In response to chronic exposure to any given stressor or the cumulation of various stressors, the inability to properly manage allostasis can be identified by the increased metabolic activity within the amygdala, augmented EPI and NOR concentrations, and dysregulated CORT concentrations at rest and in response to acute psychological stress (Pike et al., 1997; Schommer et al., 2003; Mischler et al., 2005; Tawakol et al., 2017). These responses are associated with the progressive “wear and tear” on the organism that link stress-reactivity to current CVD and future CVD events (Carroll et al., 2012; Tawakol et al., 2017; Fava et al., 2019). This shift from an effective to an inadequate (or maladaptive) physiological response is referred to as allostatic load (McEwen, 1998; McEwen, 2007). Interest in recognizing and identifying allostatic load among clinical populations has increased over the past decade. For example, Seeman et al. (2001) utilized a combination of cardiovascular, anthropometric, cholesterol, catecholamines, and other plasma biomarkers to measure the cumulative impact of allostatic load on various health outcomes and all-cause mortality. Results revealed that the progressive accumulation of allostatic load predicted decreased physical and cognitive abilities and the increased risk of CVD and all-cause mortality over a 7-year span among healthy 70–79-year-old men and women (Seeman et al., 2001). Similarly, a recent meta-analysis of 17 studies indicates that higher compared to lower indices of allostatic load contributed a 22% increase in risk for all-cause mortality and a 31% increased risk for CVD morality (Parker et al., 2022). These results suggest that the presence of repeated stress or prolonged and continuous exposure to stress, presents a challenge to allostasis and decreases the body’s ability to adapt. Thus, similar to the “exhaustion” phase proposed by Selye, the attenuated ability of the body to respond to repeated and persistent negative psychological stressors resulting in allostatic load directly contributes to the onset and progression of CVD pathology (Dar et al., 2019; Levine et al., 2021; Parker et al., 2022).

This evolution of Hans Selye’s initial conceptualization of stress has led investigators to examine factors that improve the efficiency of the adaptive response to stressors (allostasis) while minimizing over activation of these systems (allostatic load). Since psychological stress overactivity can result in many of the stress related disorders and common diseases of modern life, the identification of potential interventions that can improve an individual’s resiliency to the negative consequences of psychological stress is of increased importance. For example, psychological resiliency, in particular an individual’s perception that the stressor is a challenge worth overtaking *versus* a difficult burden to overcome, in part, can determine the magnitude of the individual’s acute response to a stressor (Lazarus, 1974). Furthermore, Felix et al. (2022) indicated that higher resiliency (e.g., family/social networks and optimism) helped mitigate against the adverse cardiovascular health outcomes associated with high allostatic load among African American men, but not women. This appraisal model suggests that when a stressor is viewed from a positive perspective or as a lesser threat, hormone secretion and cardiovascular reactivity remain lower compared to when the stressor is viewed as a burden or a larger threat to survival (Hinton et al., 1991; van Eck et al., 1996; Peters et al., 1998; Fauvel et al., 2001; Steptoe and Willemsen, 2004; Weinstein et al., 2010). Likewise, differences in perception and associated magnitude of the physiological reaction are directly linked to current and future CVD risk (Chida and Steptoe, 2010; Carroll et al., 2012), with lower CVD risk being attributed to positive perceptions of psychological stress (Dar et al., 2019; Levine et al., 2021). As such, the current level of resilience or ability to develop a more robust resiliency to a particular stressor is likely fundamental to reducing the global health burden associated with CVD health (Levine et al., 2021). Similarly, systemic environmental factors woven into the fabric of societies that disproportionally impact various populations and demographics are critical, and possibly more important, when considering strategies to reduce the adverse impact of daily psychological stress on global health.

## Psychological stress and inflammation

### General immune response to acute and chronic psychological stress

A key factor linking the etiology of psychological stress to CVD risk includes dysregulation of the immune system (Slavich, 2020). Selye first described the impact of chronic psychological stress on immune function dysregulation by describing atrophy to the thymus and lymph nodes within rodents (Selye, 1936). Similarly, it has been suggested that Hans Selye first identified the anti-inflammatory actions of glucocorticoids during the 1940s and was not provided full credit for his discoveries (Szabo et al., 2012). Nontheless, these initial observations served as the foundation for the field of PNI, a term first described by Robert Ader in 1980, and defines his and his colleagues research conducted throughout their careers (Ader and Cohen, 1975; Ader, 1980).

PNI details the role of the immune system as a mechanism linking mental and emotional stress to EPI, NOR, and CORT concentrations in health and disease (Slavich, 2020). More specifically, brief, yet persistent, challenges to allostasis, and the associated progression into allostatic load, are linked to the continual output of catecholamines and glucocorticoids and the dysregulated activation of the immune system (Marsland et al., 2017). In response to acute psychological stress, transient immune responses are commonly examined *via* changes in circulating concentrations of pro-inflammatory and anti-inflammatory cytokines. Another common measure of immune dysfunction includes identifying shifts in the distribution of immune cell types and their respective subsets examined *via* flow cytometry. Lastly, isolating innate immune cells, such as monocytes, is frequently utilized to examine changes in the sensitivity of their inflammatory signaling pathways following exposure to known inflammatory agents, such as lipopolysaccharide (LPS) or phytohaemagglutinin. In this regard, increased attention has been directed toward alterations in the psychobiological mechanisms linking acute and chronic psychological stress to dysregulation of the immune system which help explain the association of allostatic load and CVD (Acevedo and Slusher, 2017; Huang et al., 2017).

Acute models of psychological stress are currently limited to laboratory-induced or naturalistic stressors. Laboratory stressors typically occur in a controlled environment using established protocols known to activate the SAM system and HPA axis, such as the Trier Social Stress Test (TSST). In contrast, naturalistic stressors are real-life experiences, such as major life events or natural disasters, known to induce psychological stress and its associated physiological response. For both psychological and physical stimuli, acute stressors alter allostasis by increasing SAM system and HPA axis activation necessary to stimulate cardiovascular reactivity. In response to acute psychological stress, a recent meta-analysis examining over 30 publications details that among a large array of pro-inflammatory proteins, concentrations of interleukin-6 (IL-6) were also elevated within 40–50 min and remained elevated for at least 2 h following cessation of the stressor. Similar, yet more transient, increases were also exhibited for IL-1β, whereas tumor necrosis factor alpha (TNF-α), IL-2, and the anti-inflammatory cytokine IL-10 exhibited small, albeit significant, increases in circulation during the recovery periods under investigations (often up to 2 h; Marsland et al., 2017). Interestingly, the magnitude of the pro-inflammatory immune response, such as IL-6, has been shown to be greater in healthy compared to clinical populations (Marsland et al., 2017). These findings suggest that the immune response a vital component of the physiological stress response. Likewise, populations with chronic low-grade levels of systemic pro-inflammation at baseline, or rest, may exhibit a different threshold for, or altered magnitude of, an immune response compared to healthy populations that serve as a “control” group for a particular study. As such, the health and disease statuses of various populations must be considered when measuring a psychological stress-induced immune response (Furman et al., 2019).

Examining the mobilization of immune cell populations and associated subsets allows for mechanistic investigations into how various immune cell types respond to protect the host from psychological perturbations. EPI is a potent mobilizer of immune cells, characterized by the transient increase in the number and redistribution of various immune cells and their respective subsets, including the trafficking of monocytes to the vascular endothelium (Dimitrov et al., 2010). More specifically, monocytes are the predominant immune cell population responsible for pro- and anti-inflammatory cytokine production in response to acute psychological stress (Bierhaus et al., 2003). Monocytes are identified by the pattern of cell surface markers CD14 and CD16, and their inflammatory reactivity is determined by expression of the transmembrane pattern recognition receptor Toll-like receptor 4 (TLR4; Hailman et al., 1994; Slusher et al., 2018). There are three primary subsets of monocytes that are determined by the distribution patterns of the CD14 and CD16 receptors: *classical* (CD14^+bright^CD16^-^), *intermediate* (CD14^+bright^CD16^+dim^), and *non-classical* (CD14^+dim^CD16^+bright^; Passlick et al., 1989). Furthermore, *classical* monocytes express low levels of the TLR4 receptors, account for up to 80% of all monocytes, and are typically associated with IL-6 and IL-10 cytokine production (Frankenberger et al., 1996; Slusher et al., 2018). In contrast, *intermediate* and *non-classical* account for about 5% and 15% of all monocytes observed in circulation, respectively, express progressively higher levels of TLR4 receptors at the cell membrane, and are primarily responsible for IL-1β and TNF-α secretion (Frankenberger et al., 1996; Belge et al., 2002; Slusher et al., 2018). Additionally, both *intermediate* and *non-classical* monocyte subsets are preferentially mobilized in response to acute psychological stress, and thus, serve as one mechanism responsible for the increased inflammatory reactivity of monocyte observed in circulation (Dimitrov et al., 2010).

Changes in monocyte distribution patterns and inflammatory responsiveness have further enabled researchers to identify the specific molecular pathways and how they respond to acute psychological stressors. For example, although CD14 is necessary to initiate an LPS-induced inflammatory response *via* TLR4 (Wright et al., 1990; Kim and Kim, 2014), TLR4 receptors on monocytes also interact with CD16 in response to LPS stimulation at the cell surface to trigger an intracellular signaling cascade that stimulates the activation of the nuclear factor-κB (NF-κB) transcription factor (Akashi et al., 2003; Shalova et al., 2012). As a result, NF-κB undergoes a translocation from the cytoplasm to the nucleus as the primary transcription factor responsible for the synthesis of numerous inflammatory proteins in response to acute psychological stress, including IL-1β, IL-6, IL-10, and TNF-α (Bierhaus et al., 2003). Similarly, TLR4-mediated activation of NF-κB within monocytes increases activation of the Nod-like receptor pyrin containing 3 (NLRP3) inflammasome, an intracellular mulitprotein complex that is responsible for release of numerous pro-inflammatory cytokines in response to psychological stress, including IL-1β and IL-18 (Kaufmann et al., 2017). Although NLRP3-induced IL-1β expression and release has been shown to occur in an ATP-dependent manner within the hippocampus to improve symptoms of depression (Iwata et al., 2016), the release from the monocytes in peripheral circulation; and following recruitment to the brain in response to acute and psychological stress; and its role in the inflammatory response, requires additional research (Alcocer-Gómez et al., 2014). Non-etheless, Kuebler et al. (2015) further demonstrated that NF-κB binding activity increased immediately following the TSST and remained elevated for up to 1 h into recovery among healthy males aged 20–50 years. This rapid activation of the intracellular inflammatory signaling pathway was further shown to be associated with the increased expression of mRNA of various inflammatory cytokines, including IL-1β and IL-6 (Kuebler et al., 2015), which are in turn, released by immune cells as an initial mechanism to protect the host from potential tissue damage associated with the acute stress reactivity.

Marsland et al. (2017) further highlights that the *ex vivo* secretion of the pro-inflammatory cytokines IL-6 and TNF-α from isolated monocytes occurs rapidly (within about 10–30 min) and is soon followed by the secretion of IL-1β (within 30–50 min) among monocytes isolated and cultured immediately following acute psychological stress. Although the rapid release of these inflammatory proteins was observed under culture conditions and might not accurately reflect the secretion kinetics of immune cells *in vivo*, these responses are augmented greatly when stimulated in the presence of LPS, thus supporting the key functional role of the TLR4-NF-κB inflammatory pathway as initially demonstrated by Bierhaus et al. (2003). These findings suggest that the increased reactivity of pro-inflammatory pathway in combination with the transient shift towards the *intermediate* and *non-classical* subsets following an acute psychological stressor primes monocytes to elicit a robust innate immune response upon inflammatory stimulation. Likewise, these research findings are key to understanding the pathology of disease progression because the inflammatory phenotype of circulating monocytes is thought to reflect the tissue-specific microenvironment upon their migration into specific tissue sites and subsequent differentiation into resident macrophages (Bories et al., 2012).

In response to acute psychological stress, activation of the vascular endothelium results in the secretion of numerous chemokines known to attract circulating leukocytes, mainly monocytes and neutrophils, to the site of inflammation (Redwine et al., 2003; Redwine et al., 2004; Hinterdobler et al., 2021a). Similarly, vascular endothelial cells express adhesion molecules which interact with their receptors on the surface of circulating immune cells that are recruited, in part, by local NOR production within the endothelium (Hinterdobler et al., 2021a; Hinterdobler et al., 2021b). The tethering of pro-inflammatory monocytes and neutrophils and their slow rolling along the vascular endothelium eventually results in their firm adherence (arrest) and subsequent migration (extravasation) through the endothelium (Silvestre-Roig et al., 2020; Mauersberger et al., 2022). Once monocytes have transmigrated through the vascular endothelium, they differentiate into resident tissue macrophages that also exhibit a pro-inflammatory phenotype (Swirski et al., 2009; Silvestre-Roig et al., 2020). As a result, these pro-inflammatory resident macrophages, often referred to as M1-macrophages, ingest cholesterol particles, form foam cells, and can contribute, along with neutrophils, to the development of unstable plaques that prolong and exacerbate the pro-inflammatory microenvironment characteristic of atherosclerosis and coronary heart disease (Wilbert-Lampen et al., 2008; Silvestre-Roig et al., 2020; Hinterdobler et al., 2021a; Hinterdobler et al., 2021b; Gerhardt et al., 2022; Mauersberger et al., 2022).

### Interaction of catecholamines and cortisol on immune cell inflammatory pathways

The interaction of the central nervous system and the inflammatory signaling pathway in response to acute psychological stress is in part determined by the interaction of EPI and NOR with various adrenergic receptors (ADRs) located on the surface of immune cells (monocytes). For example, Bierhaus et al. (2003) demonstrated that NOR interacts with α_1_-ADRs on the surface of monocytes to increase NF-κB expression. To the contrary, EPI has been shown to interact with β_2_-ADRs to downregulate regulate TLR4 and the intracellular pro-inflammatory signaling pathway (Kizaki et al., 2008; Wang et al., 2009; Dimitrov et al., 2013; Hong et al., 2015). These interactions are important to augment the ability of immune cells to travel to various tissues and mount an immune response appropriate to protect the host in response to perceived psychological stress. Shortly after the cessation of the stressor, the HPA axis mediated release of CORT acts on the glucocorticoid receptor within the cytosol, where it subsequently translocates into the nucleus to inhibit NF-κB, downregulate the pro-inflammatory immune response, and increase transcription of the anti-inflammatory cytokine IL-10 within monocytes (Barnes, 1998; McKay and Cidlowski, 1999). The cellular sensitivity to glucocorticoids aids in returning the host to a normal physiological level once the stressor and threat to allostasis has subsided. Interestingly, glucocorticoids have been shown to enhance ATP-dependent NLRP3 expression within culture monocyte/macrophages to promote pro-inflammation, whereas more recent investigations suggest an inhibitory role within murine macrophages stimulated with LPS (Wu et a., 2020). Although the precise role of glucocorticoids remains yet to be fully elucidated, the synchronization of SAM system and HPA axis activation serve as vital mechanisms necessary to prepare and recover from a fight or flight response.

Over time, repeated exposure to acute stressors or the persistent threat to an individual’s safety or wellbeing, such as race- and obesity-related discrimination, low socioeconomic status, neighborhood safety, and/or occupational hazards, to suggest a few, contributes to the accumulation of chronic stress (Schnorpfeil et al., 2003; Nelson et al., 2007; Juster et al., 2010; Seeman et al., 2010; Tan et al., 2017; Suvarna et al., 2020). As a result, alterations of hormone concentrations at baseline and altered responsiveness to an acute psychological stressor (such as an augmented, blunted, or prolonged elevation of hormone concentrations) cause immune system dysregulation characterized by low-grade elevations in systemic inflammatory cytokine concentrations and increases in the pro-inflammatory reactivity of monocytes (Maydych et al., 2017). The prolonged exposure of monocytes to LPS increases α_1_-ADR expression on the cell surface of monocytes (Heijnen et al., 1996; Rouppe van der Voort et al., 1999; Rouppe van der Voort et al., 2000), and in parallel, β_2_-ADR sensitivity has been shown to decrease in individuals with indices of chronic levels of psychological stress and low-grade pro-inflammatory profiles (Euteneuer et al., 2012; Hong et al., 2014). Furthermore, the expression and sensitivity of glucocorticoid receptors to CORT within monocytes/macrophages is diminished in individuals with chronic elevated levels of psychological stress (Stark et al., 2001; Miller et al., 2008). Increased metabolic activity within the amygdala is also associated with increased hematopoietic stem cell activity within the bone marrow and the increased number of circulating leukocytes (Tawakol et al., 2017). These indices of chronic psychological stress contribute to the prolonged activation of the pro-inflammatory response of immune cells in circulation and within the vascular endothelium following an acute psychological stressor, and consequently, the progression of low-grade pro-inflammatory profiles in circulation and the progression of atherosclerotic inflammation involved in the development of CVD (Rodriguez et al., 2016; Tawakol et al., 2017; Kivimäki and Steptoe, 2018).

## Psychological stress adaptations to physical activity and exercise

### Physical activity improves resiliency to psychological stress and immune function

Physical activity (any action or movement of the body that requires skeletal muscle activation) and exercise (a subcategory of physical activity that is organized and planned) are two important behavioral strategies that serve as positive physical stressors to protect against the potential negative consequence of acute and chronic psychological stress (Hamer and Steptoe, 2007; Hamer, 2012; Acevedo and Slusher, 2017; Huang et al., 2017). Richard Dienstbier (1989) has proposed that “active toughening”, an adaptation to aerobic exercise training, results in the enhanced regulation of the SAM system activation (EPI and NOR concentrations and cellular ADR sensitivity) and suppression of the CORT response initiated from the HPA axis. As a result, the improved coordination of these physiological responses works in unison to increase stress tolerance, improve emotional control, and enhance immune function (Dienstbier, 1989).

Soon thereafter, Sothmann et al. (1996) provided the framework for the “cross-stressor adaptation” hypothesis. The cross-stressor adaptation hypothesis suggests that individuals with higher participation rates in routine physical activity and/or elevated cardiorespiratory fitness levels (indicated as maximal oxygen uptake; VO_2max_) experience generalized adaptations that are integrated across physiological systems. These adaptations work to make the collective actions of these systems more resilient to the negative consequences of acute stress reactivity and the hallmark health consequences of chronic stress (Sothmann et al., 1996). Similar to Dienstbier’s physiological toughening hypothesis, Sothmann et al. (1996) suggested that routine physical activity, and in particular aerobic exercise training, enhances the function and communication among the SAM system, HPA axis, and cardiovascular and muscular systems to minimize disruptions to homeostasis and allostasis during acute physical activity. Moreover, the capacity of these beneficial adaptations to physical activity to protect against the potentially harmful consequences of acute and chronic psychological stress are largely a factor of the intensity, duration, and frequency and type of activity (Mücke et al., 2018). However, just as an appropriate amount of physical activity can positively regulate indicators of psychological stress, Selye also specified that an excessive amount of exercise should be considered adverse and can contribute to the negative health consequences of chronic psychological stress (Selye, 1936). As such, physical activity and exercise recommendations suggest obtaining sufficient amounts of moderate intensity (150 min/week) or high-intensity (75 min/week) physical activity for optimal physical and psychological health while warning against excessive amounts (up to about 300 min/week) on a consistent basis (Nieman, 2020). It is evident that further investigations into the specific exercise guidelines for enhancing the cross-stressor adaptation and reducing stress related allostatic load are warranted.

In support of the physiological toughening and cross-stressor adaptation hypotheses, regular participation in physical activity is a well-known anti-inflammatory behavior, and the resultant anti-inflammatory milieu helps to lower the risk of developing CVD (Gleeson et al., 2011; Pedersen, 2017). In response to acute and chronic psychological stress, the benefits of physical activity also extend beyond buffering against adverse cardiovascular and hormonal reactivity to more favorably regulating the systemic and cellular immune responses. For example, Hamer and Steptoe (2007) initially examined the relationship between physical fitness and the systemic inflammatory cytokine response following mental stress. Results demonstrated that healthy men and women with increased performance on a submaximal cycle ergometer test exhibited lower cardiovascular reactivity (i.e., lower heart rates) and less pronounced increases in plasma IL-6 and TNF-α compared to low fit individuals during recovery from an acute psychological stressor (Hamer and Steptoe, 2007). Since this hallmark publication, increased participation in physical activity and elevated cardiorespiratory fitness levels routinely exhibit a more regulated systemic and cellular immune response to acute psychological stress as well as lower indices of inflammation when faced with chronic life stress (Puterman et al., 2018; Simpson et al., 2021).

The cellular mechanisms by which the immune system is positively regulated through physical activity participation and exercise training are vast. For example, shifts in the proportion of circulating monocytes in favor of *classical* compared to the more pro-inflammatory *intermediate* and *non-classical* subsets are typical (Timmerman et al., 2008), resulting in a lower concentration of monocytes producing pro-inflammatory proteins upon stimulation. In addition, TLR4 receptor expression is reduced, contributing to a decrease in the activation of the LPS-stimulated intracellular signaling pathway on any given subset of monocyte (Timmerman et al., 2008). Consistent with these findings, Khakroo Abkenar et al. (2019) demonstrated that although acute, high intensity exercise increases peripheral blood mononuclear cell NLRP3 gene expression as well as circulating IL-1β and IL-18 concentrations, 3-month of aerobic exercise training was sufficient to prevent these intracellular and systemic pro-inflammatory responses. Interestingly, Hong et al. (2014) has shown that monocytes exhibited increased cellular β_2_-ADR sensitivity that could play a role in downregulating the TLR4-mediated inflammatory response. Likewise, glucocorticoid receptor sensitivities within the cytosol and their ability to inhibit NF-κB activation have been shown to increase in response to regular aerobic exercise training (Duclos et al., 2003). As a result, the proportion of pro-inflammatory monocytes is decreased and the overall inflammatory sensitivity of individual monocytes to LPS is significantly lower in physically active and aerobically trained compared to inactive and untrained individuals (Timmerman et al., 2008; Slusher et al., 2018). Cumulatively, the reduced systemic and cellular inflammatory reactivity observed in physically active compared to inactive populations in response to psychological stress serve as a primary mechanism by which physical activity and exercise maintain cardiovascular health and prevent the onset of CVD during the aging process (Merino et al., 2011; Slusher et al., 2019; Simpson et al., 2021).

### Physical activity and COVID-19-associated psychological stress

In December 2019, the novel severe acute respiratory syndrome coronavirus 2 (SARS-CoV-2) emerged and spread globally. As a result, local and national stay-at home orders limited access to social connections, increased food insecurities, and prevented access to important health services, such as routine healthcare and exercise gyms/facilities (Fallon, 2020; Killgore et al., 2020; Parekh et al., 2021; Tuczyńska et al., 2021; Völker, 2023). Consequently, the role of physical activity gained attention as a safe and effective public health initiate to reduce the psychological stress associated with the initial and persistent confinement associated with lock-down orders (López-Bueno et al., 2020; Meira Jr. et al., 2020; Wolf et al., 2021). Unfortunately, physical activity tended to decrease during the initial stages of SARS-CoV-2 pandemic related restrictions (Meyer et al., 2020; Tison et al., 2020). In addition, decreased physical activity levels during the SARS-CoV-2 pandemic appeared to be more pronounced for low socioeconomic communities and children, who were out of school and void of physical education courses (Moore et al., 2020; Spencer et al., 2020). Furthermore, lack of access to safe locations for physical activity engagement may have contributed to this reduction in physical activity (Barr-Anderson et al., 2021). As a result, the SARS-CoV-2 global pandemic disproportionately impacted marginalized communities, and key avenues to make public health services more equitable were highlighted (Okonkwo et al., 2020; Hasson et al., 2022).

The SARS-CoV-2 pandemic also highlighted the unique relationship that exists between physical activity/exercise and psychological stress. For example, Salari et al. (2020) indicated that the prevalence of psychological stress, anxiety, and depression ranged from 30% to 34%, respectively, among the general public during the early months of the SARS-CoV-2 pandemic. In addition, Marashi et al. (2021) demonstrated that physical activity levels dropped in parallel with increased perceptions of psychological stress over a 6-month period among a large Canadian population. Similarly, Creese et al. (2021) suggested that indices of loneliness and decreased physical activity levels were predictors of poor mental health experienced during the SARS-CoV-2 pandemic compared to the 5-year period before the pandemic lockdown among adults 50 years of age or older. It is possible that the stress associated with the SARS-CoV-2 pandemic made it difficult to cope with the significant disruptions to daily life, as many who reported being less physically active indicated that lack of social support, increased feelings of anxiety, and lack of motivation were barriers to engagement in physical activity (Marashi et al., 2021). Upon infection, symptoms of psychological stress, including depression, anxiety, and post-traumatic stress, were more likely to be observed among COVID-19 patients compared to non-infected controls (Guo et al., 2020), as well as patients with severe compared to those with mild or less severe cases (Dong et al., 2021). More worrisome, the severity of psychological stress-related mental health issues predicted elevated plasma concentrations of the pro-inflammatory marker C-reactive protein (Guo et al., 2020). These findings suggest that the exacerbation of pro-inflammatory profiles among individuals infected with COVID-19 is associated with poorer health outcomes, including death (Lamontagne et al., 2021). Similar observations have also been documented with chronic psychological stress in patients with CVD and in patients with other low-grade pro-inflammatory conditions (Papava et al., 2022).

Active engagement in physical activity and exercise proved to be a vital mechanism for the preservations of psychological wellbeing associated with stay-at-home orders during the initial stages of the SARS-CoV-2 pandemic. For example, Wolf et al. (2021) conducted a preliminary review during the early stages of the SARS-CoV-2 pandemic and concluded that increased total time spent engaging in moderate to vigorous physical activity/exercise presented with lower depressive and anxiety-like symptoms. In support of these findings, Marashi et al. (2021) demonstrated that individuals who remained physically active or actively increased engagement in physically activity referenced the associated mental health benefits as the primary motivational factor obtained from regular physical activity participation. These findings suggest that although higher indices of psychological stress prevented regular engagement in physical activity, personal commitment to physical activity and/or increased motivation for the resultant mental health benefits supported regular physical activity engagement. As a result, consistent physical activity helped to maintain indices of mental health and attenuate levels of psychological stress during the SARS-CoV-2 pandemic. Thus, these results highlight the critical need for approaches that provided social support and direct individuals toward increased physical activity as a behavioral health strategy to maintain mental and emotional health and potentially minimize COVID-19 severity upon infection.

Lower levels of physical activity and cardiorespiratory fitness also emerged as a risk factor for infection severity, hospitalization, intensive care unit admission, and risk of premature death (Christensen et al., 2021; Sallis et al., 2021; Jimeno-Almazán et al., 2022; Nieman and Sakaguchi, 2022). A possible mechanism for this benefit of physical activity relates to the anti-inflammatory role of physical activity and exercise (Zbinden-Foncea et al., 2020; De Sousa et al., 2021). In addition to the capacity of physical activity to facilitate the inflammatory microenvironments described above, regular physical activity and exercise have also been shown to activate the ACE2 receptor within the lungs. ACE2 receptor activation stimulates the production of angiotensin 1–7 (Ang1-7) from angiotensin II (AngII) within pulmonary endothelial cells as an anti-inflammatory mechanism that helps maintain lung health (Prata et al., 2017; De Sousa et al., 2021). To the contrary, COVID-19 binds the ACE2 receptor and prevents Ang1-7 production from AngII, thereby creating an imbalance in the ratio of AngII to Ang1-7 that is associated with the COVID-19-asscociated pro-inflammatory response within the lungs (Zbinden-Foncea et al., 2020; De Sousa et al., 2021).

Another potential mechanism by which regular physical activity and exercise serve to protect against COVID-19 involves increasing the capacity of the immune system to prevent infection (Simpson et al., 2020). Although there is ongoing debate regarding the role that exercise intensity has on minimizing infection risk (Simpson et al., 2020), moderate physical activity and exercise have been shown to regulate immune function and increase the capacity of the immune system to prevent and fight against bacterial and viral infection by improving immunosurveillance (Nieman, 2020; Baker et al., 2023). Similarly, physical activity has been shown to increase vaccine effectiveness (Pascoe et al., 2014), which may play a role in decreasing anxiety associated with not just pandemic-specific vaccinations development, but also vaccine effectiveness upon administration (Bendau et al., 2021; Hallam et al., 2022). As an example, Ledo et al. (2020) previously demonstrated that neutralizing antibodies to seasonal influenza vaccination was greater among elite athletes, and Kohut et al. (2004) observed that 10 months of moderate intensity exercise increased antibody titer in response to influenza immunizations among adults 65 years of age and older. More recently, Hallam et al. (2022) has demonstrated that a single session of moderate intensity cycling (90 min), among aerobically trained individuals, can increase antibody titer without impacting vaccine-associated side effects. More recently, Batatinha et al. (2022) demonstrated that recent vaccination against COVID-19 infection did not alter the cardiorespiratory or neuroendocrine response to moderate and intense physical activity among endurance trained athletes. Therefore, these results support the increase in messaging about the benefits of acute and chronic physical activity and exercise as a behavioral mechanism to improve the immune responses to safe and effective vaccine administration (Hallam et al., 2022), which in turn, might help to lower pandemic-associated psychological stress.

## Summary

Stress today is woven into society and is important to the overall health and wellness of nearly every individual across the globe. From a broader perspective, psychological stress is an important evolutionary driver that has been integral for the survival of our species throughout history and will continue to be a critical driver of our evolution into the future. Hans Selye expanded upon science’s previous understanding of stress physiology and laid a critically important foundation for the field that contributed to relationships of acute and chronic stress and the deleterious outcomes related to cardiovascular health. Building upon his work, Bruce McEwen described the roles of allostasis and allostatic load as two mechanisms linking psychological stress to the pathophysiological progression of CVD. Simultaneously, Dienstbier and Sothmann separately described the capacity of physical activity to buffer against the harmful consequences of psychological stress by “toughening” the SAM system, HPA axis, and immune system as “cross-stressor adaptations” that maintain allostasis and prevent allostatic load. Finally, the “new normal” that has emerged during the ongoing SARS-CoV-2 global pandemic has provided a lens through which scientists can once again appreciate the tremendous contributions of Han Selye’s work on stress and health, and how his contributions have impacted worldwide public health policy.
